# OCT4 and SOX2 Specific Cytotoxic T Cells Exhibit Not Only Good Efficiency but Also Synergize PD-1 Inhibitor (Nivolumab) in Treating Breast Cancer Stem-Like Cells and Drug-Resistant Breast Cancer Mice

**DOI:** 10.3389/fonc.2022.781093

**Published:** 2022-03-24

**Authors:** Wei Peng, Liang Chang, Wenqiang Li, Yanan Liu, Min Zhang

**Affiliations:** ^1^ Department of General Surgery, Guangdong Provincial People's Hospital Zhuhai Hospital (Zhuhai Golden Bay Center Hospital), Zhuhai, China; ^2^ Department of Intensive Care Unit, Guangdong Provincial People's Hospital Zhuhai Hospital (Zhuhai Golden Bay Center Hospital), Zhuhai, China; ^3^ Department of General Surgery, Cixi People’s Hospital, Ningbo, China

**Keywords:** OCT4 and SOX2, cytotoxic T lymphocytes, PD-1 inhibitor, breast cancer stem-like cells, drug-resistance breast cancer

## Abstract

**Purpose:**

This study aimed to investigate the effect of OCT4&SOX2 specific cytotoxic T lymphocytes (CTLs) plus programmed cell death protein-1 (PD-1) inhibitor (nivolumab) on treating breast cancer stem-like cells (BCSCs) *in vitro* and drug-resistance breast cancer (DRBC) mice *in vivo*.

**Methods:**

In total, 160 breast cancer patients were enrolled following the immunofluorescence assay to detect tumor OCT4 and SOX2 expressions. CD154-activated B cells were co-cultured with CD8^+^ T cells (from breast cancer patients) in the presence of OCT4&SOX2 peptides, CMV pp65 peptides (negative control), and no peptides (normal control). MCF7-BCSCs were constructed by drug-resistance experiment and sphere-formation assay, then DRBC mice were constructed by planting MCF7-BCSCs. Subsequently, different doses of OCT4&SOX2 CTLs and PD-1 inhibitor (nivolumab) were used to treat MCF7-BCSCs and DRBC mice.

**Results:**

OCT4 and SOX2 correlated with poor differentiation, more advanced stage, and worse prognosis in breast cancer patients. *In vitro*, OCT4&SOX2 CTLs with effector-target ratio (ETR) 5:1, 10:1 and 20:1 presented with increased cytotoxic activity compared to CMV pp65 CTLs with ETR 20:1 (negative control) and Control CTLs with ETR 20:1 (normal control) on killing MCF7-BCSCs. Besides, PD-1 inhibitor (nivolumab) improved the cytotoxic activity of OCT4&SOX2 CTLs against MCF7-BCSCs in a dose-dependent manner. *In vivo*, OCT4&SOX2 CTLs plus PD-1 inhibitor (nivolumab) decreased tumor volume and tumor weight while increased tumor apoptosis rate compared to OCT4&SOX2 CTLs alone, PD-1 inhibitor (nivolumab) alone, and control.

**Conclusion:**

OCT4&SOX2 CTLs exhibit good efficiency and synergize PD-1 inhibitor (nivolumab) in treating BCSCs and DRBC.

## Introduction

Breast cancer is the most commonly diagnosed malignancy and a leading cause of cancer-associated mortality in females worldwide. According to the Global Cancer Statistics 2018 Report (1), the new breast cancer cases were approximately 2.09 million, with 0.63 million cancer deaths in 2018. Although considerable improvements have been made in the early diagnosis and novel treatments (such as targeted drugs and individualized therapy plans) and decreased mortality rates by ≤30% in the past 25 years, the prognosis of breast cancer is still unsatisfactory (2, 3). Besides, multiple factors are causing the poor prognosis in patients with breast cancer, including drug resistance, subsequent disease relapse, and metastasis (4, 5). Breast cancer stem-like cells (BCSCs) are a small proportion of stem-like breast cancer cells and critical factors for disease relapse and metastasis that exhibit self-renewal properties and the ability to differentiate into various types of breast cancer cells, which are highly resistant to multiple drugs in breast cancer (6, 7). Therefore, exploring novel treatments to diminish BCSCs is of great importance for improving the prognosis of breast cancer patients.

Recent improvements in immunotherapy have achieved a new milestone in treating numerous cancer types, including breast cancer (8, 9). As an immunotherapeutic option, cytotoxic T lymphocytes (CTLs) can selectively induce apoptosis in target cells *via* perforin/granzyme and Fas/tumor necrosis factor-mediated mechanisms, further serving as potential candidates for the treatment of cancers (10, 11); in fact, several previous studies have revealed the potential of CTLs to target specific types of cancer stem cells (CSCs), such as colon lung CSCs (12, 13). However, the inhibitory effect of CTLs depends mainly on their numbers, unimpaired functionality, and the antigen recognition of targeted cells. To the best of our knowledge, no previous study has reported the use of CTLs to target BCSCs (13, 14). Octamer-binding transcription factor 4 (OCT4) and sex-determining region Y-box 2 (SOX2) are considered to be promising BCSC markers, whose expression is closely associated with multi-drug resistance in breast cancer. Therefore, it was hypothesized that OCT4- and SOX2-specific CTLs (OCT4&SOX2 CTLs) might target and effectively destroy BCSCs (15–17). In addition, inhibitors of programmed cell death protein-1 (PD-1) and its ligand (PD-L1) are important immunotherapy options. For instance, attenuating PD-1 results in T cells apoptosis, energy, and exhaustion; PD-L1-mediated prevention of CTL-mediated lysis and improved anti-tumor immune responses (18, 19). Thus, we further deduced that using OCT4&SOX2 CTLs with nivolumab (a PD-1 inhibitor) may synergistically treat BCSCs.

In the present study, OCT4 and SOX2 expressions were detected in breast cancer tissues to explore their correlations with tumor features and prognosis in breast cancer patients. Subsequently, we investigated the effect of OCT4&SOX2 CTLs combined with nivolumab on treating both BCSCs *in vitro* and mice with drug-resistant breast cancer (DRBC) *in vivo*.

## Materials and Methods

### Patients

In total, 160 patients aged from 29 to 79 years with breast cancer who underwent surgical resection between January 2013 and December 2015 were retrospectively reviewed. The inclusion criteria were as follows: i) Diagnosed with primary breast cancer; ii) aged >18 years; iii) complete clinicopathological data before surgery and complete follow-up data were accessible; iv) tumor tissue and paired-adjacent tissues were accessible, and v) had not received neoadjuvant therapy. The Ethics Committee of our hospital approved the present study, and all patients (or their guardians) provided written informed consent or oral agreement with tape recording.

### Tissue Samples and OCT4/SOX2 Expression Measurement

Tumor and paired-adjacent tissues were obtained from the Specimen House of our Hospital, and the expression of OCT4 and SOX2 was detected using immunofluorescence assays. Briefly, formalin-fixed, paraffin-embedded tissues were cut into 6-µm slices, deparaffinized, and subsequently rehydrated. The slices were then transparentized using polybutylene terephthalate and soaked in a solution of 1% bovine serum albumin plus 0.1% Triton for 30 min. Antigen retrieval was performed in a microwave using 3% hydrogen peroxide, after which the slices were blocked with 10% goat serum (cat. no. 16210064, Gibco, USA) and incubated with Rabbit anti-OCT4 (1:1,600; cat. no. 2750; CST, USA) and Mouse anti-SOX2 (1:400; cat. no. 4900; both Cell Signaling Technology, Inc.) antibodies at 4°C overnight. Then, the slices were washed in SuperBlock™ T20 (TBS) Blocking Buffer (cat. no. 37536; Thermo Fisher Scientific, USA) and incubated with an anti-rabbit IgG AlexaFluor^®^ 488-conjugated antibody (1:500; cat. no. 2975) and an anti-mouse IgG AlexaFluor^®^ 594-conjugated antibody (1:500; cat. no. 8527; both Cell Signaling Technology, Inc.). Finally, the slices were washed with buffer, stained with 2-(4-aminophenyl)-6-indolecarbamidine dihydrochloride (DAPI), and then covered with coverslips. The expression of OCT4 and SOX2 was semi-quantitatively assessed under an inverted fluorescence microscope (X400) (Nikon Corporation) using the following intensity criteria: i) 0, no staining; ii) 1, weak but detectable staining; iii) 2, moderate or distinct staining; and iv) 3, intense staining. A histological score (HSCORE) was derived using the following formula: HSCORE = ΣPi(i+1), where i represents the intensity score, and Pi is the corresponding cell percentage. An HSCORE of 0.7 was used as the cut-off point to divide the samples into OCT4 and SOX2 high- and low-expression groups. In addition, OCT4 and SOX2 expression in tumor and paired-adjacent tissues were also detected by immunohistochemical (IHC) staining, using rabbit anti-OCT4 antibody (1:250; cat. no. ab200834; Abcam, UK) and rabbit anti-SOX2 antibody (1: 50; cat. no. ab93689; Abcam, UK) antibodies. The IHC process was performed strictly according to the standard protocol. Besides, OCT4 and SOX2 IHC score was calculated, referring to the staining intensity score multiplying the staining density score. The staining intensity was scored as 0 (no staining), 1 (week staining), 2 (moderate staining), 3 (intense staining), and the staining density was scored as 0 (negative), 1 (≤25%), 2 (26-50%), 3 (51-75%), 4 (≥76%). The total IHC score ranged from 0 to 12. IHC score >3 was defined as high expression, while an IHC score of ≤3 was defined as low expression.

### Collection of Clinical and Follow-Up Data

Pre-surgery patient clinicopathological data were collected from the Electronic Medical Records Management System at our Hospital and included tumor, node, metastasis (TNM) stage, and pathological stage, as well as estrogen receptor (ER), progesterone receptor (PR), and human epidermal growth factor receptor 2 (HER-2) statuses. Patients were followed up until June 2017, and overall survival (OS) time was calculated from the time of surgical resection to the time of death. Besides, expression data of OCT4 and SOX2 and their effects on survival in breast cancer patients were derived from the Protein Atlas Database (https://www.proteinatlas.org/).

### Establishment of CD154-Activated B Cells

CD154-activated B cells were generated as previously described (12). Briefly, i) NIH3T3 cells overexpressing CD154 (CD154^+^) were prepared by Shanghai GenePharma Co., Ltd., using lentiviral transfection. The cells were cultured in 90% Dulbecco’s modified Eagles medium (DMEM; cat. no. A4192101) supplemented with 10% fetal bovine serum (FBS; cat. no. 12483020) (both Gibco; Thermo Fisher Scientific, Inc.), 100 U/ml penicillin and 100 μg/ml streptomycin at 37˚C (5% CO_2_, 95% air). ii) A total of 150 ml peripheral blood was obtained from 30 patients newly diagnosed with breast cancer (5 ml per patient) after the ethics approval of our hospital and the signed informed consents, and the peripheral blood mononuclear cells (PBMCs) were isolated; CD19^+^ cells (primary B cells) were obtained using CD19^+^ magnetic beads (cat. no. 130-050-301; Miltenyi Biotec GmbH) and then cultured in 88% Iscove’s Modified Dulbecco’s Medium (cat. no. 12440061; Gibco; Thermo Fisher Scientific, Inc.) supplemented with 10% FBS, 2% insulin, transferrin and selenium (ITS) additive, 100 U/ml IL-2 (cat. no. SRP3085; Sigma-Aldrich; Merck KGaA), 40 ng/ml IL-17 (cat. no. SRP3080; Sigma-Aldrich; Merck KGaA) and one µg/ml cyclosporine A (cat. no. HY-B0579; MedChemExpress). iii) CD154^+^ NIH3T3 cells were then used as the feeder layer to activate the primary B cells for three weeks. To determine the antigen-presenting ability of CD154-activated B cells, the proportions of the CD80^+^, HLA-ABC^+^, and CD86^+^ cell populations were measured by flow cytometry (FCM), with primary B cell cultures alone (without CD154^+^ NIH3T3 cells) as the control group (**Supplementary Figures 1A, B**). The monoclonal antibodies used for FCM were CD80-FITC (cat. no. 11-0809-41), HLA-ABC-PE (cat. no. 25-9983-42), and CD86-APC (cat. no. 17-0862-82; all Invitrogen; Thermo Fisher Scientific, Inc.).

### Establishment of OCT4&SOX2 CTLs

CTLs with both OCT4 and SOX2 antigen presentation (OCT4&SOX2 CTLs) were constructed to partially reference the methods described in a previous study (12). Briefly: i) Peripheral blood samples were obtained from 30 newly diagnosed patients with breast cancer after the Ethics Approval of our hospital and the signed informed consents, to a total volume of 300 ml (10 ml per patient); the PBMCs were isolated, and an accelerated co-cultured dendritic cell (acDC) assay was conducted in the presence of OCT4 and SOX2 peptides, according to previously described methods (20). ii) CD3^+^CD8^+^ cells (CD8^+^ T cells) were isolated using a CD8^+^ T Cell Isolation Kit (cat. no. 130-045-201; Miltenyi Biotec, German); and iii) The CD8^+^ T cells were cultured with CD154-activated B cells at a 2:1 ratio in the presence of 200 IU/ml IL-2, five ng/ml IL-17 (both Sigma-Aldrich; Merck KGaA) and one µg/ml CD3 antibody (cat. no. ab135372; Abcam) for one week. CD8^+^ T cells were then isolated and cultured with CD154-activated B cells at a 5:1 ratio with 200 IU/ml IL-2, five ng/ml IL-17, and one µg/ml CD3 antibody for another week. OCT4&SOX2 CTLs were isolated and used for further experimentation with BCSCs: i) To determine the effect of acDCs on CD8+ T cell expansion, PBMCs were subjected to an acDC assay (acDC group) [with un-assayed PBMCs as the control group (Control)] and subsequently double-stained with anti-CD3 FITC and anti-CD8 APC monoclonal antibodies (cat. nos. 11-0031-82 and MCD0827, respectively; both Invitrogen; Fisher Scientific, Inc.). CD8^+^ T cells (CD3^+^CD8^+^) were then detected using FCM. ii) Cytomegalovirus (CMV) pp65 CTLs and control CTLs were also prepared; the former was prepared using CMV pp65 peptides instead of OCT4&SOX2 peptides (using the methods above) and were not peptide-primed. BankPepitide Biotech Company constructed the peptides, and the sequences were as follows: OCT4, DVVRVWFCNRRQKGK; SOX2, PWRTMDASERGRLLYKLADLIERD, and CMV pp65, ALVPMVATV.

### Establishment of BCSCs

The MCF7 human breast cancer cell line was purchased from the Cell Resource Center of Shanghai Institute of Life Sciences, Chinese Academy of Sciences (Shanghai, China) (cat. no. TCHu-74), and cultured in 90% MEM (cat. no. 12492013; Gibco; Thermo Fisher Scientific, Inc.) with 10% FBS at 37˚C (5% CO_2_, 95% air). BCSCs were then prepared by establishing drug-resistant MCF7 cells (R-MCF7), followed by a sphere formation assay. R-MCF7 cells were constructed as follows: MCF7 cells were treated with 2.4 μmol/l Adriamycin (cat. no. D1515; Sigma-Aldrich; Merck KGaA) for 72 h, and then cultured in Adriamycin-free medium for a further 72 h. Cellular proliferation was assessed using the Cell Counting Kit-8 (CCK-8; cat. no. E606335; Sangon Biotech Co., Ltd.). The process was repeated until the effects of Adriamycin were affected on cell proliferation were no longer detectable. The entire treatment process was then repeated using 4.8 μmol/l Adriamycin. MCF7 cells treated with 2.4 μmol/l Adriamycin only (non-resistant cells) served as the positive control.

Subsequently, the R-MCF7 cells were subjected to a sphere formation assay as previously described (21): R-MCF7 cells were cultured in serum-free DMEM/F12 (cat. no. A4192002) supplemented with 2% B27 (cat. no. 0080085SA; both Gibco; Thermo Fisher Scientific, Inc.), 20 ng/ml epidermal growth factor (cat. no. E5036), 20 ng/ml basic fibroblast growth factor (cat. no. GF003AF) and 4 μg/ml heparin (cat. no. H3149-500KU-9; all Sigma-Aldrich; Merck KGaA) for ten days; the resulting spheres were isolated and served as MCF7 BCSCs in subsequent experiments. To verify the successful establishment of the MCF7 BCSCs, the expression of common CSC markers (CD44, CD133, OCT4, and SOX2) was determined and compared between MCF7 BCSCs and normal MCF7 cells using reverse transcription-quantitative PCR (RT-qPCR) and western blotting.

Besides, after MCF7 BCSCs were established *via* escalated-dose adriamycin treatment followed by sphere formation assay in MCF7 cells, they presented with increased CD44, CD133, OCT4, and SOX2 expressions compared to normal MCF7 cells (**Supplementary Figures 2A-G**).

### Measurement of the Effect of OCT4&SOX2 CTLs on Killing BCSCs

Control CTLs with an effector-target ratio of 20:1, CMV pp65 CTLs with an effector-target ratio of 20:1, and OCT4&SOX2 CTLs with effector-target ratios of 1:1, 5:1, 10:1 and 20:1 were used to treat MCF7 BCSCs, and divided into six corresponding groups, respectively. Meanwhile, MCF7 BCSCs without treatment were used as Blank control. Briefly, MCF7 BCSCs were labeled with CFSE as target cells, and the corresponding CTLs with different effector-target ratios were added for 24 h; dead cells were labeled with propidium iodide (PI), and FCM was performed to detect the CFSE^+^PI^+^ and CFSE^+^PI^-^ cell populations (1x10^4^ events per group). The cytotoxic activity of CTLs was calculated as follows: CFSE^+^PI^+^/(CFSE^+^PI^+^ + CFSE^+^PI^-^) x 100%.

### Effect of OCT4&SOX2 CTLs Plus Nivolumab on Killing BCSCs

Various concentrations of nivolumab (0, 2, 4, 8, 16, and 32 nM) (cat. no. A2002; Selleck Chemicals) and OCT4&SOX2 CTLs with an effector-target ratio of 20:1 were used to treat MCF7 BCSCs for 24 h; cell proliferation was detected busing the CCK-8 assay. OCT4&SOX2 CTLs, as well as BCSCs alone, were used as controls. ii) OCT4&SOX2 CTLs with an effector-target ratio of 20:1 (alone) and OCT4&SOX2 CTLs with an effector-target ratio of 20:1 plus 32 nM nivolumab were used to treat MCF7 BCSCs, and subsequently divided into two correspondingly groups, respectively. MCF7 BCSCs were labeled with CFSE as target cells, and the corresponding CTLs were added 24h. The cytotoxic activity of the CTLs was determined using FCM as aforementioned.

### Establishment of a DRBC Mouse Model

Specific pathogen-free nude mice (age, 4-6 weeks; weight, 15-20 g) were purchased from Shanghai Laboratory Animal Center (Shanghai, China). After anesthesia by 26 mg/kg propofol *via* intravenous tail injection, 2x10^6^ MCF7 BCSCs were injected into the right dorsal flanks of the mice, and magnetic resonance imaging (MRI) was performed to assess tumor establishment one week post-implantation. The mice were then used as the DRBC mouse model for subsequent experiments. The animal experiments were conducted according to the “Guideline of Animal Experiment” and “Code for the Care and Use of Animals for Scientific Purposes” statement of our Hospital, and the Animal Ethics Committee approved the protocol.

### Effect of OCT4&SOX2 CTLs Plus Nivolumab to Treat DRBC Mice

After confirmation of tumor generation, 24 DRBC mice were randomly divided into the following four groups for five weeks of treatment: Control group, OCT4&SOX2 CTL group, nivolumab group, and OCT4&SOX2 CTL plus nivolumab group. Each group was administered with the following treatments twice a week: i) OCT4&SOX2 CTL group, 2x10^6^ OCT4&SOX2 CTLs; ii) nivolumab group, 200 μg nivolumab; iii) OCT4&SOX2 CTL plus nivolumab group, 2x10^6^ OCT4&SOX2 CTLs and 200 μg nivolumab; and iv) Control group, an equal volume of saline. The 5-week experimental duration was set (22–25). After treatment initiation, the following examinations/experiments were performed: i) animal health and behavior were monitored every week, and their welfare considerations were taken, such as minimizing the suffering/distress and good housing conditions.; ii) Survival was calculated from the time of treatment initiation to the time of disease-related death; iii) tumor were observed and their volumes calculated at weeks 0 (W0), 1 (W1), 2 (W2), 3 (W3), 4 (W4) and 5 (W5) using MRI; iv) after five weeks of treatment, all mice were euthanized by cervical dislocation according to AVMA Guidelines for the Euthanasia of Animals, then the tumors were isolated and weighed, while tumors from mice that had died during treatment were immediately harvested. The tumor inhibition rate was calculated as follows: Tumor inhibition rate = (tumor weight of control group - tumor weight of experimental group)/tumor weight of control group x 100%; and iv) tumor tissues were fixed using 10% formaldehyde solution at four °C for 24 h and embedded in paraffin for terminal deoxynucleotidyl transferase-mediated dUTP-biotin nick end labeling assays (TUNEL) (cat. no. T2190; Beijing Solarbio Science & Technology Co., Ltd.), were performed according to the instructions of the manufacturers. Furthermore, 6 out of 24 mice were dead due to tumor progression, 18 out of 24 mice were euthanized at the end time point of the study for sample collection. Meanwhile, OCT4 and SOX2 expressions in mice tumor samples were also detected using an IHC assay.

### RT-qPCR

Total RNA was extracted from the cells using TRIzol^®^ Reagent (cat. no. 15596026; Invitrogen; Thermo Fisher Scientific, Inc.) and was reverse transcribed into cDNA using the PrimeScript™ RT reagent Kit (cat. no. RR047A; Takara Bio, Inc.),. qPCR was then performed using the SYBR^®^ Green Real-time PCR Master Mix (cat. no. QPK-201; Toyobo Life Science). The thermo-cycling conditions were 95 °C pre-denature 60 s for one cycle, 95 °C denature 15s, and 61°C anneal 30 s for 40 cycles. The expression levels of the target mRNAs were calculated using the 2^-ΔΔCt^ method with GAPDH as the internal reference (26). The primer sequences were as follows: CD44 forward, 5’-ACATCCTCACATCCAACACCTC-3’ and reverse, 5’-CCTCCTGAAGTGCTGCTCCT-3’; CD133 forward, 5’-GCTGCTTGTGGAATAGACAGAATG-3’ and reverse, 5’-GAAGGACTCGTTGCTGGTGAAT-3’; OCT4 forward, 5’-AAGCGATCAAGCAGCGACTA and reverse, 5’-CAGAGTGGTGACGGAGACAG-3’; SOX2 forward, 5’-ATGTCCCAGCACTACCAGAGC-3’ and reverse, 5’-GTGTGGATGGGATTGGTGTTCTC-3’; and GAPDH forward, 5’-GAGTCCACTGGCGTCTTCAC-3’ and reverse, 5’-ATCTTGAGGCTGTTGTCATACTTCT-3’.

### Western Blotting

Total protein was extracted from the cells with RIPA Lysis and Extraction Buffer (cat. no. 89901; Thermo Fisher Scientific, Inc.). The concentration was determined and adjusted using a Bicinchoninic Acid Protein Assay kit (cat. no. BCA1-1KT; Sigma-Aldrich; Merck KGaA). A total of 20 μg protein was then fractionated by SDS-PAGE using 10% Bis-Tris Protein Gels (cat. no. NP0335BOX; Invitrogen; Thermo Fisher Scientific, Inc.) and transferred to a polyvinylidene fluoride membrane (cat. no. C583; EMD Millipore). The membrane was subsequently blocked in 5% skim milk at 37°C for 90 mins and incubated with rabbit anti-human CD44 (1:2,000; cat. no. ab216647), CD133 (1:2,000; cat. no. ab226355), CT4 (1:1,000; cat. no. ab109183), SOX2 (1:1,000; cat. no. ab93689) and GAPDH (1:1,000; cat. no. ab128915) antibodies (all Abcam) at 4˚C overnight. The membrane was then incubated with a goat anti-rabbit IgG H&L (HRP) secondary antibody (1:4,000; cat. no. ab6721; Abcam) at room temperature for one h. Finally, the blot was treated with High sensitive ECL luminescence reagent (cat. no. D601039; Sangon Biotech Co., Ltd.) and exposed to x-ray film.

### Statistical Analysis

All statistical analyses were performed using SPSS Software 21.0 (IBM Corp) and presented using GraphPad Prism Software 6.01 (GraphPad Software, Inc.). The data are primarily presented as counts (percentage) or the mean ± standard deviation. Comparisons between two paired groups were determined using the McNemar test; comparisons between two individual groups were determined by t-test or the χ^2^ test. Comparisons among ≥3 separate groups were determined using one-way ANOVA followed by Dunnett’s multiple comparisons test. The χ2 test determined the association between two paired groups, and OS was exhibited as Kaplan-Meier (K-M) curves and compared using the log-rank test. P<0.05 was considered to indicate a statistically significant difference.

## Results

### Association of OCT4 and SOX2 Expressions With Clinicopathological Characteristics and Prognosis in Breast Cancer Patients

**Figure 1:**
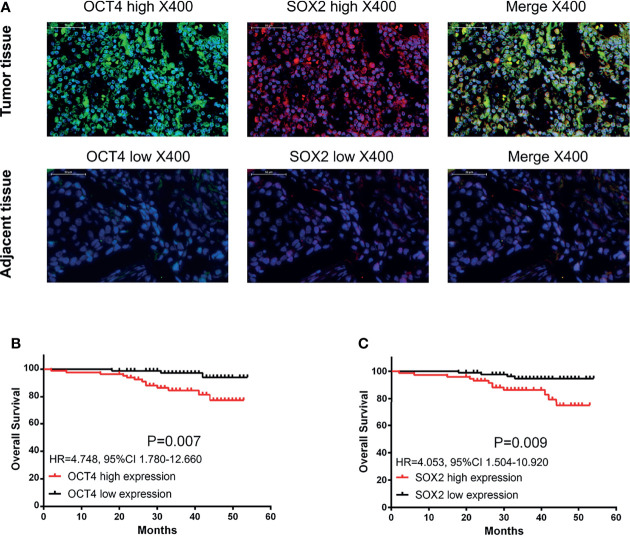
OCT4 and SOX2 expressions by IF assay and their correlation with prognosis in breast cancer patients. Examples of OCT4 and SOX2 expressions by IF assay in tumor tissue and adjacent tissue **(A)**. Correlation of tumor OCT4 and SOX2 expression with overall survival **(B, C)**.

*Via* IF assay, OCT4 and SOX2 expressions increased in breast cancer tissues compared with paired-adjacent non-tumor tissues (**Figure 1A** and **Table 1**). Besides, tumor OCT4 expression was associated with higher T stage, TNM stage, and poor differentiation, while SOX2 expression was correlated with elevated T stage, N stage, TNM stage, and poor differentiation (**Table 2**). Notably, both OCT4 [hazard ratio (HR)=4.748, 95%confidence interval (CI)=1.780-12.660] and SOX2 high expressions (HR=4.053, 95%CI=1.504-10.920) were associated with worse OS (**Figures 1B, C**).

**Table 1:** OCT4 and SOX2 expressions in tumor tissue and paired adjacent tissue.

Items	OCT4 expression		SOX2 expression	
	High	Low	High	Low
Tumor tissue (n/%)	82 (51.2)	78 (48.8)	72 (45.0)	88 (55.0)
Paired adjacent tissue (n/%)	53 (33.1)	107 (66.9)	49 (30.6)	111 (69.4)
P value	0.002		0.009	

Comparison was determined by McNemar test. OCT4, octamer-binding transcription factor 4; SOX2, sex determining region Y-box 2.

**Table 2:** Correlation of OCT4 and SOX2 expressions with clinicopathological features.

Clinicopathological parameters		OCT4 expression		P value	SOX2 expression		P value
		High	Low		High	Low	
Age (n/%)	<60 years	55 (52.9)	49 (47.1)	0.573	46 (44.2)	58 (55.8)	0.790
	≥60 years	27 (48.2)	29 (51.8)		26 (46.4)	30 (53.6)	
T stage (n/%)	T1	22 (45.8)	26 (54.2)	<0.001	17 (35.4)	31 (64.6)	<0.001
	T2	46 (47.4)	51 (52.6)		41 (42.3)	56 (57.7)	
	T3	14 (93.3)	1 (6.7)		14 (93.3)	1 (6.7)	
N stage (n/%)	N0	38 (48.1)	41 (51.9)	0.129	31 (39.2)	48 (60.8)	0.049
	N1	21 (44.7)	26 (55.3)		19 (40.4)	28 (59.6)	
	N2	21 (65.6)	11 (34.4)		20 (62.5)	12 (37.5)	
	N3	2 (100.0)	0 (0.0)		2 (100.0)	0 (0.0)	
TNM stage (n/%)	I	10 (52.6)	9 (47.4)	0.024	6 (31.6)	13 (68.4)	0.006
	II	46 (44.2)	58 (55.8)		41 (39.4)	63 (60.6)	
	III	26 (70.3)	11 (29.7)		25 (67.6)	12 (32.4)	
Differentiation (n/%)	Well	17 (48.6)	18 (51.4)	<0.001	12 (34.3)	23 (65.7)	0.013
	Moderate	45 (43.7)	58 (56.3)		44 (42.7)	59 (57.3)	
	Poor	20 (90.9)	2 (9.1)		16 (72.7)	6 (27.3)	
ER (n/%)	Negative	33 (56.9)	25 (43.1)	0.281	31 (53.4)	27 (46.6)	0.105
	Positive	49 (48.0)	53 (52.0)		41 (40.2)	61 (59.8)	
PR (n/%)	Negative	41 (56.9)	31 (43.1)	0.192	37 (51.4)	35 (48.6)	0.142
	Positive	41 (46.6)	47 (53.4)		35 (39.8)	53 (60.2)	
HER-2 (n/%)	Negative	55 (51.4)	52 (48.6)	0.956	50 (46.7)	57 (53.3)	0.532
	Positive	27 (50.9)	26 (49.1)		22 (41.5)	31 (58.5)	

Comparison was determined by Chi-square test. OCT4, octamer-binding transcription factor 4; SOX2, sex determining region Y-box 2; TNM, tumor-node-metastasis; ER, estrogen receptor; PR, progesterone receptor; HER-2, human epidermal growth factor receptor 2.

In addition, OCT4 and SOX2 expressions were also detected by IHC assay (**Supplementary Figure 3A**), which exhibited that both OCT4 high expression and SOX2 high expression linked with worse OS (**Supplementary Figures 3B, C**)

### Cytotoxic Activity of OCT4&SOX2 CTLs Against BCSCs

**Figure 2:**
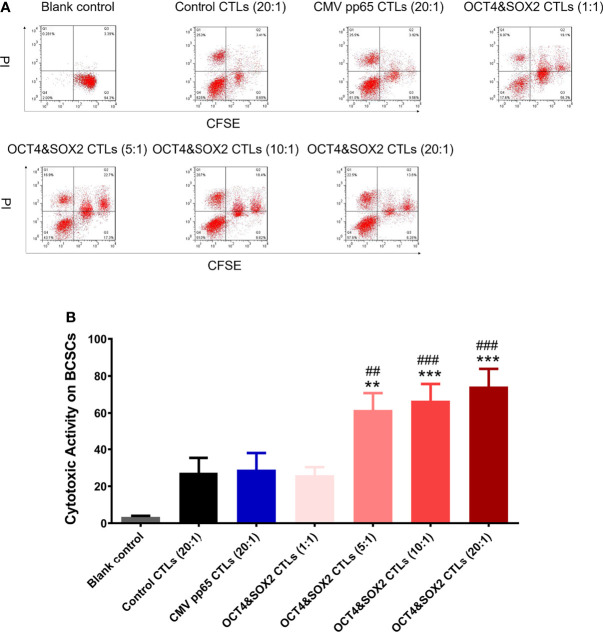
Cytotoxic activity of OCT4&SOX2 CTLs on killing BCSCs. Cytotoxic activity against MCF7 BCSCs among Blank control, OCT4&SOX2 CTLs, CMV pp65 CTLs (negative control) and Control CTLs (normal control) groups **(A, B)**. ** meant *P*<0.01 compared with Control CTLs group; *** meant *P*<0.001 compared with Control CTLs group; ## meant *P*<0.01 compared with CMV pp65 CTLs group; ### meant *P*<0.001 compared with CMV pp65 CTLs group.

OCT4&SOX2 CTLs (effector-target ratio 5:1, 10:1, 20:1), but not OCT4&SOX2 CTLs (effector-target ratio 1:1) exhibited superior cytotoxic activity over CMV pp65 CTLs (negative control) and Control CTLs (normal control) (both effector-target ratio 20:1) against MCF7 BCSCs (**Figures 2A, B**). In addition, the cytotoxic activity of OCT4&SOX2 CTLs was dose-dependent with effector-target ratio 20:1 presenting best effect towards MCF7 BCSCs (**Figures 2A, B**).

### PD-1 Inhibitor (Nivolumab) Enhanced the Effect of OCT4&SOX2 CTLs on BCSCs

**Figure 3:**
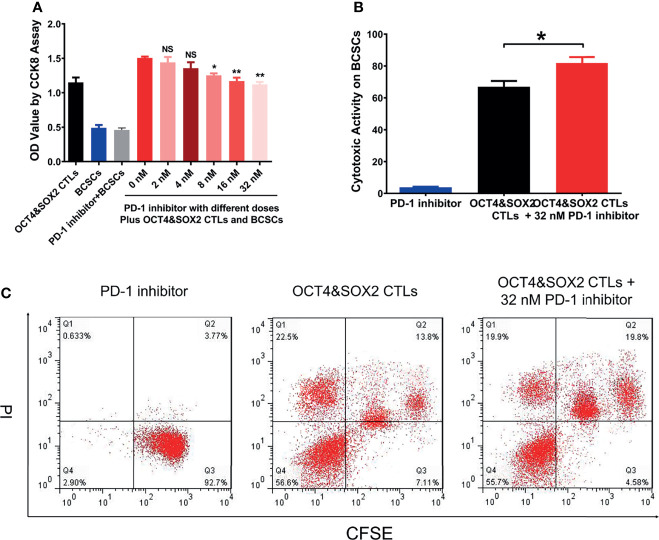
Combined effect between OCT4&SOX2 CTLs and PD-1 inhibitor (nivolumab) on treating BCSCs. Cell viability *via* CCK8 by OCT4&SOX2 CTLs plus different doses of PD-1 inhibitor (nivolumab) in BCSCs **(A)**. Direct cytotoxic activity *via* FCM assay among PD-1 inhibitor (nivolumab) group, OCT4&SOX2 CTLs plus PD-1 inhibitor (nivolumab) group and OCT4&SOX2 CTLs group **(B, C)**. NS, meant *P*>0.05; * meant *P*<0.05; ** meant *P*<0.01.

PD-1 inhibitor (nivolumab) improved the cytotoxic activity of OCT4&SOX2 CTLs against MCF7 BCSCs in a dose-dependent manner by the CCK-8 assay (**Figure 3A**). Furthermore, the FCM assay also observed that PD-1 inhibitor (nivolumab) enhanced the cytotoxic activity of OCT4&SOX2 CTLs against MCF7 BCSCs (**Figures 3B, C**).

### Synergistic Effect of OCT4&SOX2 CTLs and PD-1 Inhibitor (Nivolumab) on Treating DRBC Mice

**Figure 4:**
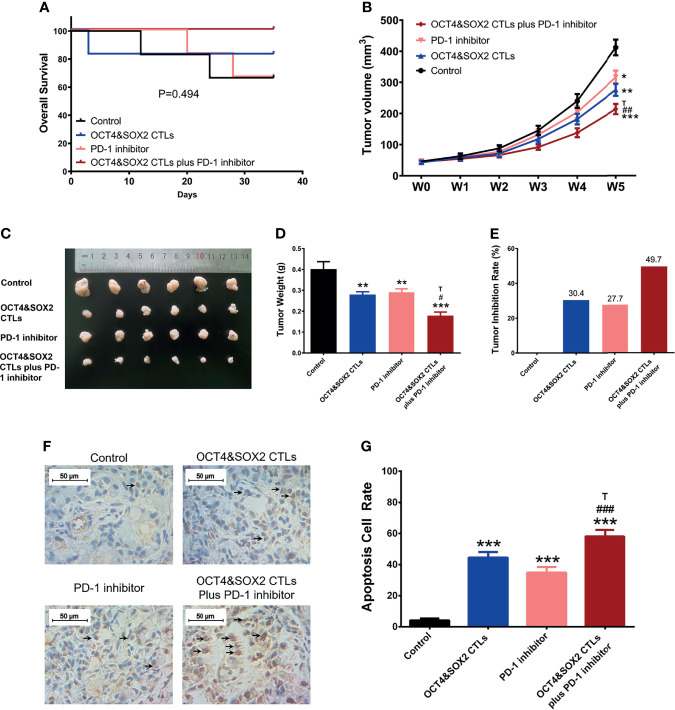
*In vivo* assessment of OCT4&SOX2 CTLs plus PD-1 inhibitor (nivolumab) on treating DRBC mice. Survival profile **(A)**, tumor volume **(B, C)**, tumor weight **(D)**, tumor inhibition rate **(E)**, tumor apoptosis rate **(F, G)** among OCT4&SOX2 CTLs plus PD-1 inhibitor (nivolumab) group, OCT4&SOX2 CTLs alone group, PD-1 inhibitor (nivolumab) alone group, and Control group. Blank arrows indicate some examples of TUNEL positive cells. * meant *P*<0.05 compared to Control group; ** meant *P*<0.01 compared to Control group; *** meant *P*<0.001 compared to Control group. # meant *P*<0.05 compared to PD-1 inhibitor (nivolumab); ## meant *P*<0.01 compared to PD-1 inhibitor (nivolumab); ### meant *P*<0.001 compared to PD-1 inhibitor (nivolumab); T meant *P*<0.05 compared to OCT4&SOX2 CTLs group.

**Figure 5:**
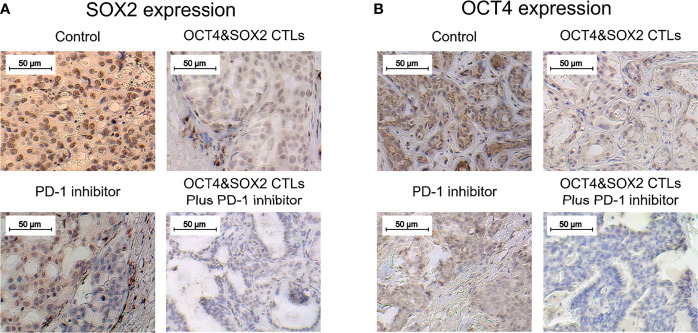
OCT4 and SOX2 expressions in DRBC mice after treatment. SOX2 expression **(A)** and OCT4 expression **(B)** by IHC assay among OCT4&SOX2 CTLs plus PD-1 inhibitor (nivolumab) group, OCT4&SOX2 CTLs alone group, PD-1 inhibitor (nivolumab) alone group, and Control group.

No difference was observed in survival among OCT4&SOX2 CTLs plus PD-1 inhibitor (nivolumab), PD-1 inhibitor (nivolumab) alone, OCT4&SOX2 CTLs alone, and Control groups (**Figure 4A**). Both PD-1 inhibitor (nivolumab) alone and OCT4&SOX2 CTLs alone decreased tumor volume and tumor weight, increasing tumor apoptosis rate (**Figures 4B–G**). Furthermore, regarding reduced tumor volume, tumor weight, and elevated tumor apoptosis rate, it was worthy to note that OCT4&SOX2 CTLs plus PD-1 inhibitor (nivolumab) showed better effects compared to PD-1 inhibitor (nivolumab) alone and OCT4&SOX2 CTLs alone (**Figures 4B–G**). In addition, it was also observed that OCT4 and SOX2 protein expressions were both highest in the Control group, followed by the PD-1 inhibitor group, then OCT4&SOX2 CTLs group, and the lowest in OCT4&SOX2 CTLs plus PD-1 inhibitor group (**Figures 5A, B**).

## Discussion

As two key transcription factors involved in the tumor progression and differentiation, OCT4 and SOX2 enhance tumor cell stemness and serve as CSC markers in various cancer types, including breast cancer (27, 28). OCT4 has been reported to increase drug resistance to gefitinib and cancer stemness in lung cancer cells and promote tumor progression and CSC proliferation in a breast cancer mouse model (29, 30). Furthermore, OCT4 expression correlates with a higher pathological grade and poor patient prognosis in several cancers (27, 31, 32). SOX2 has been illustrated to increase cellular proliferation, colony formation, and metastasis by promoting WNT/β-catenin signaling and epithelial-mesenchymal transition (EMT) and downregulating AMP-activated protein kinase/mTOR signaling in breast cancer cells. SOX2 also activates Tregs by interacting with C-C motif chemokine 1, leading to breast cancer cell stemness (33–35). In the present study, OCT4 and SOX2 expression was upregulated in patients with breast cancer and was associated with an increased TNM stage, poor differentiation, and poor prognosis. The possible explanations are as follows: i) OCT4 and SOX2 are well-known oncogenetic factors, which reflect the hyper-proliferation of various types of cancer cells, including breast cancer; therefore, higher levels of OCT4 and SOX2 may be expressed in breast cancer tissues with malignant capacity, compared with adjacent non-cancerous tissues; ii) OCT4 and SOX2 may promote breast cancer cell proliferation, migration, invasion, EMT and stemness by upregulating the activity of multiple oncogene pathways, which subsequently contribute to increased TNM stage and poor differentiation; and iii) OCT4 and SOX2 might indirectly and directly affect prognosis *via* their associations with advanced tumor features and by reducing breast cancer drug sensitivity, respectively.

As immune cells are pivotal in the antitumor response, CTLs directly target tumor cells for lysis, promoting selective apoptosis of the target cells *via* perforin/granzyme and Fas/tumor necrosis factor-mediated mechanisms (10, 11). An adaptive cellular immunotherapeutic approach (transfusing *in vitro*-induced tumor-specific CTLs into patients) is reported to be effective for treating several different types of malignancy (especially drug-resistant cancers), including breast cancer. However, the success of CTL treatment relies heavily on antigen recognition of target cancer cells and sufficient CTL numbers. Traditionally, antigen-presenting cells (APCs) [dendritic cells (DCs) isolated from the peripheral blood of patients] were used to stimulate the maturation of naïve T lymphocytes and to promote antigen presentation; however, this approach is limited by cost, the large volume of blood required and the lack of DC amplification efficiency (36). Thus in the present study, CD154-activated B cells were prepared (used as APCs) using CD154^+^ NIH3T3 cells as the feeder layer to activate primary B cells isolated from patients (12); these CD154-activated B cells presented with good antigen-presenting ability. Since peripheral B cells are more accessible to culture and amplify *in vitro*, these data further support using CD154-activated B cells as potential APCs for CTL priming. To obtain sufficient CTL numbers, an acDC assay was used to activate and amplify T lymphocytes, and the CD154-activated B cells were used to activate CD8^+^ T cells according to a previous study (12). This provided a solid foundation for subsequent experimentation.

Numerous studies have revealed that tumor-specific CTLs possess favorable cytotoxicity towards various types of cancer cell; in the presence of glypican-3 (GPC3), GPC3-specific CTLs diminish hepatocellular carcinoma cells (37), and melanoma-associated antigen 3 (MAGE-3)-specific CTLs inhibits the proliferation of bladder cancer cells and the growth of tumor xenografts in nude mice (38). Furthermore, mucin 1-specific CTLs show the potential to treat several cancer types, such as pancreatic cancer, melanoma, and colon cancer (39).

However, a few reports disclose the effects of tumor-specific CTLs on CSC killing. The reduction of CSCs is the most critical issue in cancer-associated drug resistance, metastasis, and relapse (40). A previous study reported that the adoptive transfer of centrosomal protein 55-specific CTLs inhibits tumor growth and the proliferation of colon cancer stem-like cells (13); another study revealed that OCT4 and SOX2-specific CTLs possess adequate killing capacity towards both lung cancer and lung cancer stem cells (12). To the best of our knowledge, there are currently no reports on the effect of tumor-specific CTLs on the treatment of BCSCs. In the present study, OCT4&SOX2 CTLs were hypothesized to exhibit good cytotoxic activity against BCSCs, based on the following observations: i) The expression of OCT4 and SOX2 was found to be associated with poor differentiation and worse prognosis in patients with breast cancer; ii) OCT4 and SOX2 were reported to be valuable markers of CSCs, including BCSCs; iii) targeting OCT4 and SOX2 resulted in anti-tumor effects towards CSCs, and iv) a previous study demonstrated that OCT&SOX2 CTLs effectively target and destroy lung cancer stem cells. OCT4&SOX2 CTLs also exerted cytotoxic activity towards BCSCs in the present study and were shown to function in a dose-dependent manner. There are two possible explanations for this: i) OCT4 and SOX2 are common BCSC markers. Thus OCT4&SOX2 CTLs efficiently recognize BCSCs and induce their apoptosis, and ii) the killing effect of CTLs depends on their sufficient numbers, therefore the cytotoxic activity of OCT4&SOX2 CTLs is dose-dependent. Encouragingly, a previous study novelly explores a multiepitope peptide vaccine made up of immunodominant epitopes of SYCP1 and ACRBP antigens as prophylactic melanoma vaccine, which shows good antigen-presenting ability and apparent therefore anti-cancer activity (41); the same research group also applies the co-immunization with the DNA and peptide vaccines containing SYCP1 and ACRBP epitopes to treat murine triple-negative breast cancer model, which also exhibits satisfied anti-tumor efficiency (42).

It was also noted that there were four cell populations after CTLs treatment in BCSCs; we thought the four populations included: target cancer cells with apoptosis or non-apoptosis (the first population on the right side); target cancer cells binding with CTLs with apoptosis or non-apoptosis (the second population on the right side); CTLs with apoptosis (Q1 population); CTLs without apoptosis (Q4 population). Meanwhile, it was also observed that PD-1 inhibitor alone rarely affects BCSCs viability, which could be explained by the fact that PD-1 inhibitor exhibits an anti-tumor effect *via* regulating cytotoxic T cells instead of tumor cells directly.

PD-1/PD-L1 signaling is critical to enhanced tumor progression, metastasis, and drug resistance and acts as a crucial component of tumor immunosuppression; this includes inhibiting T lymphocyte activation and enhancing tumor cell immune tolerance, leading to tumor cell immune escape (19, 43). As a first-generation immune checkpoint inhibitor, nivolumab may improve the immune response and selectively restore a tumor-induced immune deficiency in the tumor microenvironment (44). Furthermore, nivolumab reportedly promotes the antitumor effect of several other immunotherapeutics; for instance, a previous study revealed a synergistic effect between nivolumab and a cytotoxic T lymphocyte-associated antigen four blockade (9D9) (another representative first-generation four immune checkpoint inhibitor) in melanoma treatment (45). Another study illustrated that nivolumab enhances the antitumor effect of recombinant IL-21 in several cancer mouse models (46). Based on the data above, it was hypothesized that nivolumab could improve the cytotoxic activity of OCT4&SOX2 CTLs towards BCSCs. The present study revealed that nivolumab enhanced the cytotoxicity of OCT4&SOX2 CTLs against BCSCs in a dose-dependent manner and validated the synergistic effect between nivolumab and OCT4&SOX2 CTLs on BCSC killing. However, only the CCK-8 was performed to evaluate proliferation; thus alternative proliferation assays (such as Brdu or Edu) may be required in the future. *In vivo* experiments were also performed, revealing that OCT4&SOX2 CTLs may be effective for treating DRBC. Most importantly, nivolumab and OCT4&SOX2 CTLs exhibited a synergistic effect when treating DRBC mice. Possible explanations include nivolumab: i) Increasing CTL activation and decreasing immune tolerance, thus resulting in reduced tumor immune escape; ii) selectively restoring tumor-induced immune deficiency in the tumor microenvironment; iii) facilitating the accumulation of CTLs at tumor sites by increasing homing and persistence mechanisms; and iv) directly promoting tumor cell death. Meanwhile, OCT4 and SOX2 protein expressions were both highest in the control group, followed by the PD-1 inhibitor group, then OCT4&SOX2 CTLs group, and the lowest in OCT4&SOX2 CTLs plus PD-1 inhibitor group. The explanations were: PD-1 inhibitor alone repressed tumor growth generally regardless of the OCT4 and SOX2 expressions on tumor cells, therefore it less affected the OCT4 and SOX2 expressions; while OCT4&SOX2 CTLs alone or in combination with PD-1 inhibitor could selectively repress OCT4/SOX2 expressed tumor cells, therefore, their expressions were lower. Furthermore, it could be mentioned: it was our neglect that we focused on the treatment effect of OCT4&SOX2 specific CTLs plus PD-1 inhibitor but did not assess the safety. Besides, the experiments were finished, so the safety profile could not be considered retrospectively, which needs to be explored in future studies.

In conclusion, the present study reveals that using OCT4&SOX2 CTLs plus PD-1 inhibitor (nivolumab) may serve as a therapeutic approach for BCSCs and DRBC.

## Data Availability Statement

The original contributions presented in the study are included in the article/**Supplementary Material**. Further inquiries can be directed to the corresponding authors.

## Ethics Statement

The studies involving human participants were reviewed and approved by Zhuhai Golden Bay Center Hospital. The patients/participants provided their written informed consent to participate in this study. Written informed consent was obtained from the individual(s) for the publication of any potentially identifiable images or data included in this article.

## Author Contributions

YL and MZ contributed to the conception. WP, LC, and WL contributed to the experiment. WP, YL, and MZ contributed to data acquisition and analysis. All authors drafted the manuscript. All authors revised the manuscript. All authors read and approved the final manuscript.

## Conflict of Interest

The authors declare that the research was conducted in the absence of any commercial or financial relationships that could be construed as a potential conflict of interest.

## Publisher’s Note

All claims expressed in this article are solely those of the authors and do not necessarily represent those of their affiliated organizations, or those of the publisher, the editors and the reviewers. Any product that may be evaluated in this article, or claim that may be made by its manufacturer, is not guaranteed or endorsed by the publisher.

## Supplementary Material

Supplementary Figure 1 Antigen-presenting ability of CD154-activated B cells. CD80^+^, HLA-ABC^+^ and CD86^+^ cell proportions between CD154-activated B cells and control B cells **(A, B)**. ** meant *P*<0.01.

Supplementary Figure 2 Establishment of MCF7 BCSCs. R-MCF7 cells were established by Adriamycin repeated treatment until no effect of Adriamycin on cell proliferation was observed, and original MCF7 cells treated by Adriamycin was served as positive control **(A)**. Then sphere formation assay was performed and spheres were isolated and served as MCF7 BCSCs **(B)**. The increased CD44, CD133, OCT4 and SOX2 protein and mRNA expressions in MCF7 BCSCs than normal MCF7 cells validated the successful establishment of MCF7 BCSCs **(C-G)**. NS, meant *P*>0.05; ** meant *P*<0.01; *** meant *P*<0.001.

Supplementary Figure 3 OCT4 and SOX2 expressions by IHC assay and their relation with prognosis. Examples of OCT4 and SOX2 expressions by IHC assay in tumor tissue and in adjacent tissue **(A)**. Correlation of tumor OCT4 and SOX2 high expression by IHC assay with overall survival **(B, C)**.
