# Supplementary figures and images for: OCT4 and SOX2 Specific Cytotoxic T Cells Exhibit Not Only Good Efficiency but Also Synergize PD-1 Inhibitor (Nivolumab) in Treating Breast Cancer Stem-Like Cells and Drug-Resistant Breast Cancer Mice
Source: Front Oncol. 2022 Mar 24;12:781093. doi: 10.3389/fonc.2022.781093 (PMC8987438; doi:10.3389/fonc.2022.781093)

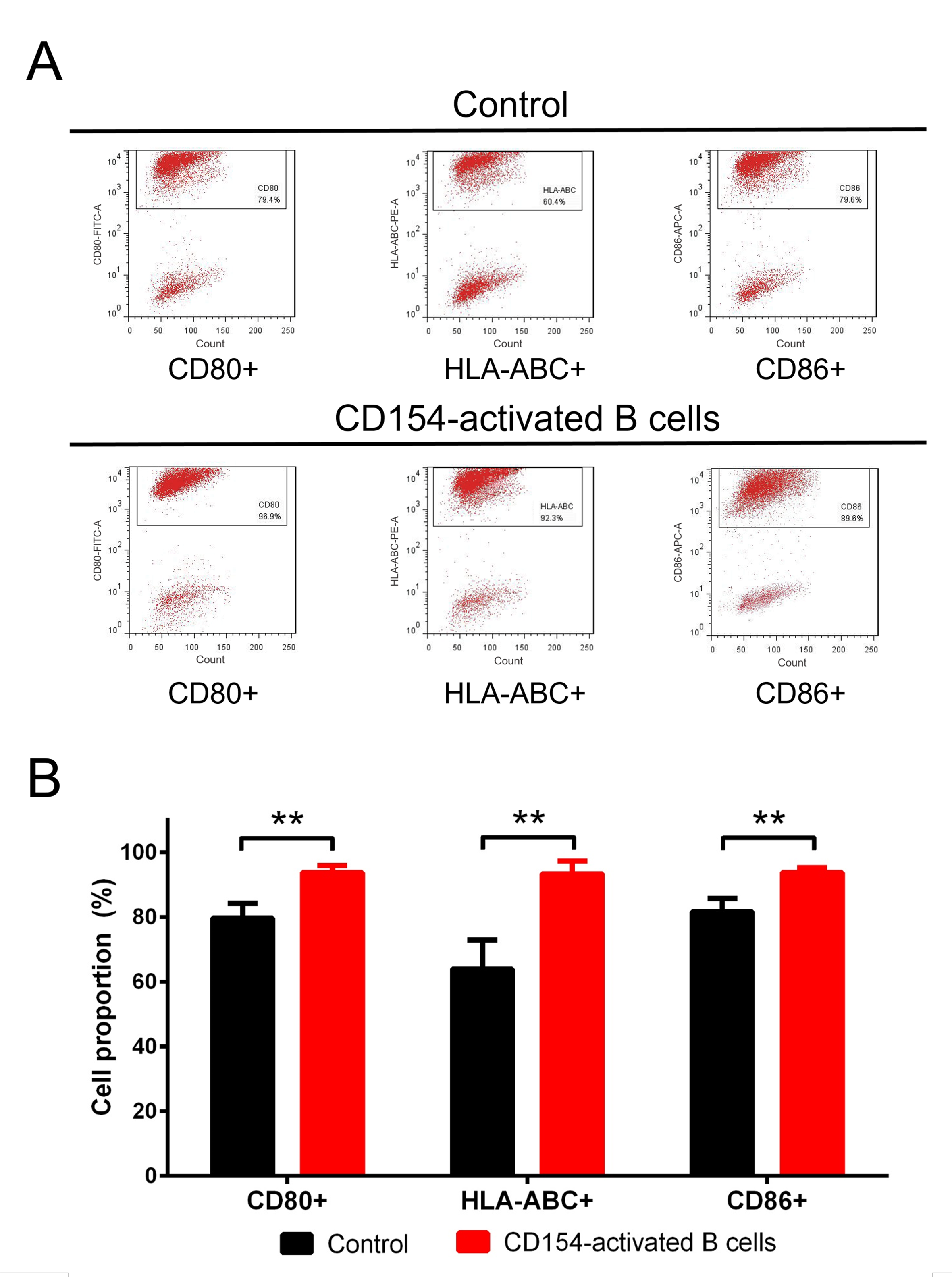

Supplement: Supplementary Figure 1 — Antigen-presenting ability of CD154-activated B cells. CD80+, HLA-ABC+ and CD86+ cell proportions between CD154-activated B cells and control B cells (A, B). ** meant P<0.01. [file Image_1.tif]

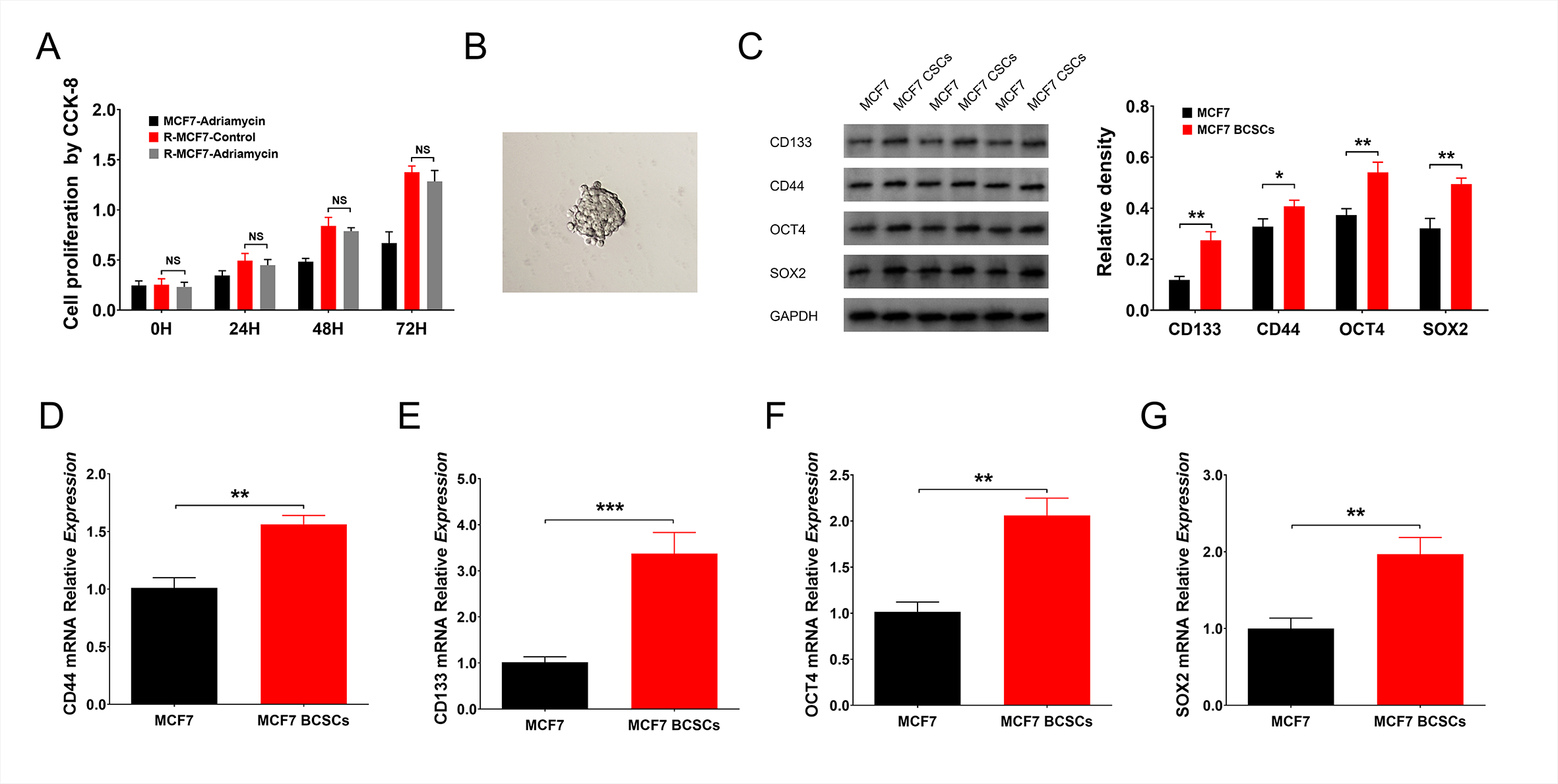

Supplement: Supplementary Figure 2 — Establishment of MCF7 BCSCs. R-MCF7 cells were established by Adriamycin repeated treatment until no effect of Adriamycin on cell proliferation was observed, and original MCF7 cells treated by Adriamycin was served as positive control (A). Then sphere formation assay was performed and spheres were isolated and served as MCF7 BCSCs (B). The increased CD44, CD133, OCT4 and SOX2 protein and mRNA expressions in MCF7 BCSCs than normal MCF7 cells validated the successful establishment of MCF7 BCSCs (C-G). NS, meant P>0.05; ** meant P<0.01; *** meant P<0.001. [file Image_2.tif]

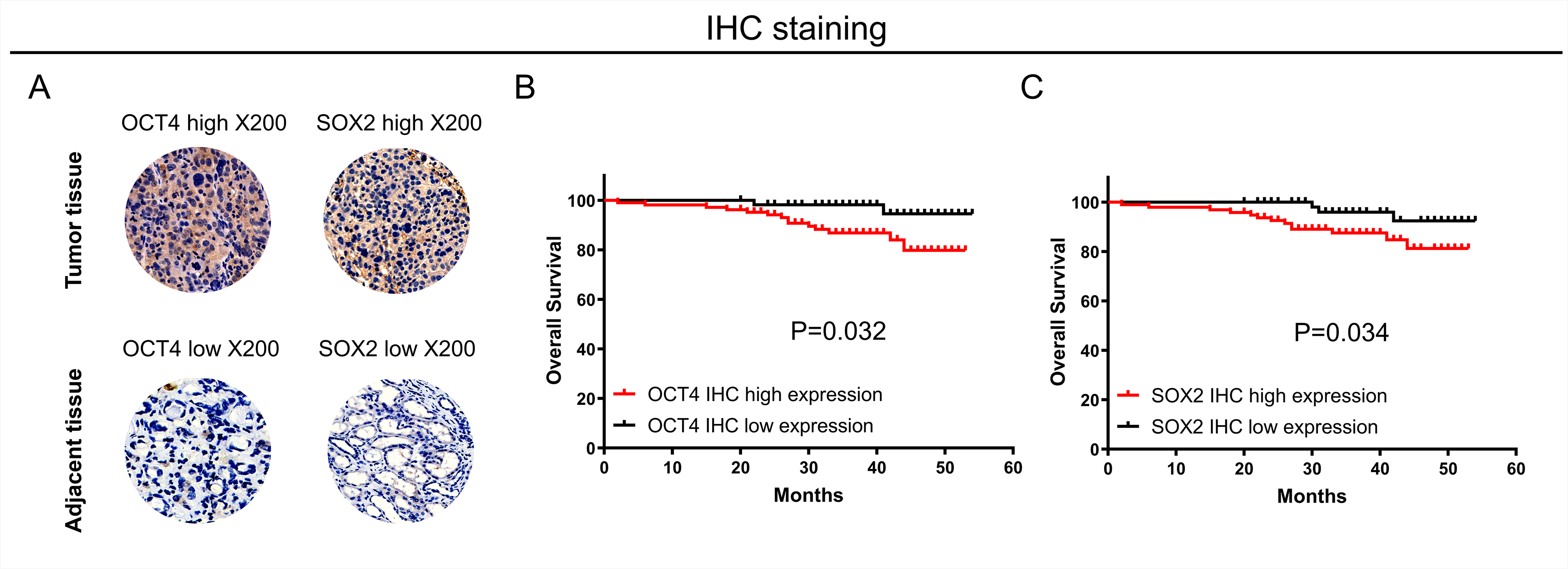

Supplement: Supplementary Figure 3 — OCT4 and SOX2 expressions by IHC assay and their relation with prognosis. Examples of OCT4 and SOX2 expressions by IHC assay in tumor tissue and in adjacent tissue (A). Correlation of tumor OCT4 and SOX2 high expression by IHC assay with overall survival (B, C). [file Image_3.tif]
